# Drought-Stressed Tomato Plants Trigger Bottom–Up Effects on the Invasive *Tetranychus evansi*

**DOI:** 10.1371/journal.pone.0145275

**Published:** 2016-01-06

**Authors:** Miguel G. Ximénez-Embún, Félix Ortego, Pedro Castañera

**Affiliations:** Department of Environmental Biology, Centro de Investigaciones Biológicas, CSIC, Madrid, Spain; Hainan University, CHINA

## Abstract

Climate change will bring more drought periods that will have an impact on the irrigation practices of some crops like tomato, from standard water regime to deficit irrigation. This will promote changes in plant metabolism and alter their interactions with biotic stressors. We have tested if mild or moderate drought-stressed tomato plants (simulating deficit irrigation) have an effect on the biological traits of the invasive tomato red spider mite, *Tetranychus evansi*. Our data reveal that *T evansi* caused more leaf damage to drought-stressed tomato plants (≥1.5 fold for both drought scenarios). Mite performance was also enhanced, as revealed by significant increases of eggs laid (≥2 fold) at 4 days post infestation (dpi), and of mobile forms (≥2 fold and 1.5 fold for moderate and mild drought, respectively) at 10 dpi. The levels of several essential amino acids (histidine, isoleucine, leucine, tyrosine, valine) and free sugars in tomato leaves were significantly induced by drought in combination with mites. The non-essential amino acid proline was also strongly induced, stimulating mite feeding and egg laying when added to tomato leaf disks at levels equivalent to that estimated on drought-infested tomato plants at 10 dpi. Tomato plant defense proteins were also affected by drought and/or mite infestation, but *T*. *evansi* was capable of circumventing their potential adverse effects. Altogether, our data indicate that significant increases of available free sugars and essential amino acids, jointly with their phagostimulant effect, created a favorable environment for a better *T*. *evansi* performance on drought-stressed tomato leaves. Thus, drought-stressed tomato plants, even at mild levels, may be more prone to *T evansi* outbreaks in a climate change scenario, which might negatively affect tomato production on area-wide scales.

## Introduction

Agricultural production faces the challenge to produce more food while constrained by a number of biotic and abiotic factors. Climate change is predicted to produce an increase in temperature and drought events in the next decades, especially in the mid-continental and Mediterranean climate areas where they are expected to be more frequent and intense [1]. Drought is by far the leading environmental stress in agriculture that limits the global productivity of major crops by directly reducing plant potential yield [2], but also by indirectly influencing their interactions with biotic factors, as a consequence, playing a critical role on the world´s food security.

Drought stress has been historically advocated as one key factor for herbivorous outbreaks [3, 4]. Yet, the relationship between arthropod outbreaks and drought is not consistent, depending on the timing, intensity and water stress phenology [5] and on the feeding guild that the herbivore belongs to [6]. It is widely accepted that drought stress triggers significant alterations in plant biochemistry and metabolism [7] that may alter the physiology of the host plant and modify the nutritional values, affecting herbivore performance [8]. There are several hypotheses concerning the response of the plant to drought stress and how herbivores adapt to those changes [5, 9, 10]. Drought induces metabolic changes in the plant, such as increased levels of free sugars and free essential amino acids, which according to the “Plant stress hypothesis” causes the plant to have a higher nutritional value for herbivores [6, 10, 11], and can play an important role in herbivore outbreaks [12, 13]. In contrast, drought is also associated with a reduction in growth and an increase in defense compounds making the plant less suitable for herbivores according to the “Plant Vigor Hypothesis” [9]. The resulting performance of phytophagous arthropods on drought-stressed plants will then depends on the access they have to an optimal balance of nutrients in the plant according to their feeding habit [5], and their adaptation to plant defense compounds according to their grade of specialization [14].

Climate change is expected to increase the incidence of water shortage in semi-arid environments. Then, deficit irrigation scheduling, yielding mild and moderate drought, might help to improve the efficiency with which water is used in major crops, such as tomato, widely cultivated in semi-arid regions. The tomato agro-ecosystem is threatened by a few major key pests, such as spider mites, and many minor or secondary pests [15]. The red tomato spider mite, *Tetranychus evansi* Baker & Pritchard was first recorded in Brazil, and has emerged as a serious invasive agricultural pest in invaded areas such as Africa and Europe [16]. In last decade, it has been considered one of the most important pests of solanaceous crops in Africa, causing high yield lossess in tomato in some African regions [17]. This species has been reported as highly tolerant to hot and dry conditions. As a result of climate change, the Mediterranean basin is the most threatened area for the potential spread of *T*. *evansi* [18]. In fact, outbreaks have been recorded in Europe, particularly around the Mediterranean basin where *T*. *evansi* has spread significantly in the last decades [18]. The high invasive potential of *T*. *evansi* and the severity of damage have prompted its addition to the alert list of the European and Mediterranean Plant Protection Organization [19].

When feeding on tomato leaves, *T*. *evansi* was found to suppress anti-mite plant defenses by down-regulating the expression of genes involved in the regulation of secondary metabolites and defense proteins, increasing the fitness of these mites in the absence of competitors [20, 21]. However, water deficit stress in tomato plants had been reported to increase some of these plant defenses, such as protease inhibitors and the oxidative enzymes polyphenol oxidases and peroxidases [6, 22, 23]. On the other hand, water deficit stress can elicit accumulations of nutrients in tomato plants. Among such nutrients, free amino acids, which are known to be instrumental in the maintenance of host plant osmotic balance, and free sugars are usually detected at elevated levels, and can therefore contribute to improve mite performance [24]. Moreover, free proline, a nonessential amino acid for arthropods that accumulates in most drought-stressed plants, has been reported to be a feeding stimulant for many phytophagous species [4]. However, there is little information on the combined effects of both biotic (mites) and abiotic (drought) stress on the tomato plant physiology and on how these changes influence *T*. *evansi* performance.

This study represents an attempt to establish if mild or moderate drought stress (simulating two scenarios of deficit irrigation) induces physiological changes (i.e. nutritional values and chemical defenses) in tomato plants, and if they have an effect on *T*. *evansi* behavior, development and physiology. This holistic approach provides insight into the bottom-up effects that may influence mite performance on drought-stressed tomato plants and the implications for outbreak risks of *T*. *evansi*, which might strongly affect tomato production on area-wide scales.

## Materials and Methods

### Plant material and mites rearing

A colony of *T*. *evansi* collected in Beausoleil (South of France) was provided by Dr. Maria Navajas (CBGP, France). Mites were maintained on detached tomato leaves (*Solanum lycopersicum* L., cv. Moneymaker) placed on ventilated plastic cages (22x30x15 cm) for about 30 generations. The petiole of the leaves was in contact with a thin layer of water in the bottom of the cages to avoid mites escaping and to maintain the leaf turgor. Tomato plants were grown from seeds in 40 wells trays. Plants with 3 expanded leaves were transferred to 2.5 liters pots (diameter: 16 cm, height: 15 cm) (Maceflor, Valencia, Spain) filled with 600 g of Universal growing medium `Compo sana´ (Compo GmbH, Münster, Germany) and watered to saturation. The mite colony and the tomato plants were maintained on a climate room at 25°C±1°C, 50±5% relative humidity and a 16 h light/8 h dark photoperiod.

### Drought stress regime and experimental design

**Fig 1:**
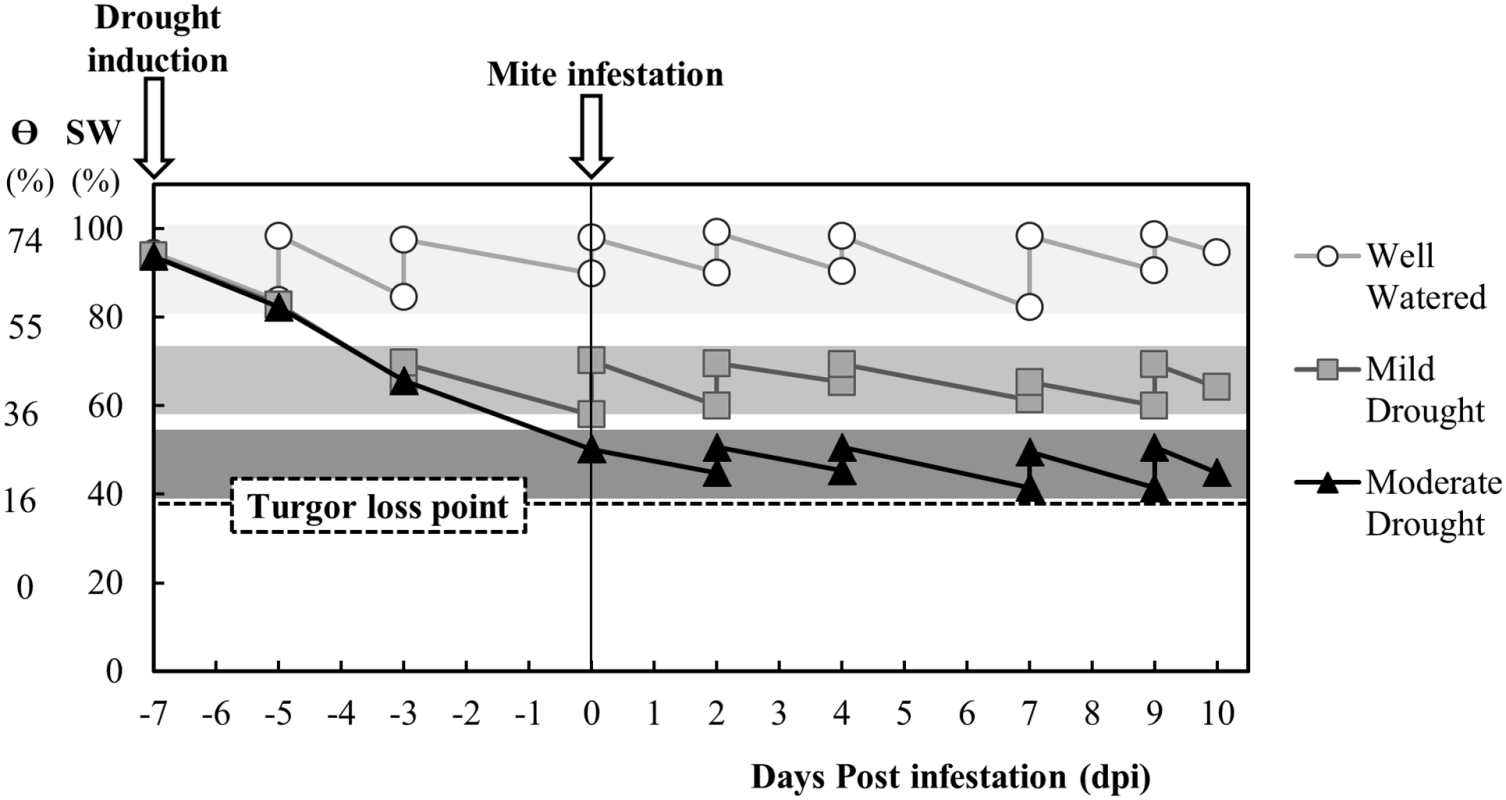
Water stress status (well watered, mild and moderate drought) of tomato plant. Drought induction was started 7 days before mite infestation and was continued until the end of the experiment [10 days post infestation (dpi)]. It is expressed as percentages of saturation weight (SW) and volumetric soil water content (Ɵ). The turgor loss point was established as 40% of SW.

Water stress status of single tomato plants was quantified gravimetrically by frequent recording of pot weights (balance BSH 6000, PCE Iberica, Tobarra, Spain), and transpired water was replaced by intermittently watering them such that soil water content was maintained within a narrow range (Fig 1). Measurements of soil water status was determined by using two parameters: percentage of saturation weight [SW = (weight of 100% water saturated soil−soil weight)/weight of 100% water saturated soil]; and the volumetric soil water content [Ɵ = (soil weight−soil dry weight)/(H_2_O density*soil volume); soil dry weight was determined by drying 600 g of soil on stove at 70°C for 3 days]. Tomato plants with 4–5 expanded leaves were watered every 2–3 days to maintain the SW in the range of 80–95% (corresponding to Ɵ between 55–74%) for the control plants. We imposed two water stress regimes, defined as mild and moderate, by stopping irrigation for 4 or 7 days, respectively, and thereafter watering every 2–3 days to maintain the SW in the range of 60–73% (Ɵ = 36–50%) for mild stress and between 45–53% (Ɵ = 16–36%) for the moderate stress (Fig 1). Both water stress regimes were over the wilting point associated with severe drought stress [25] that was established at 40% of SW for Moneymaker in our experimental conditions.

Mild and moderate stressed plants were coupled with well-watered (control) plants in two independent experiments. In each experiment, plants were divided into eight different groups combining two different treatments: Drought stress (control or drought) and *T*. *evansi* infestation (infested or no infested); and two sampling times (4 or 10 days post infestation = dpi). Spider mites performance and leaf damage was assessed in both mild and moderate drought stress experiments. Chemical and biochemical analysis of plant material was only performed for moderate drought stress (the utmost applied).

Tomato plants (6–7 expanded leaves) were infested when steady stress conditions were reached (Fig 1) by placing 8 spider mites females on each of the two leaflets next to the apical one of the leaves 3, 4 and 5 (48 mites per plant). Mites were collected from the laboratory colony using a vacuum pump D-95 (Dinko S.A., Barcelona, Spain) with a sucking power of 10–50 mmHg connected to a modified Eppendorf. All plants were confined with a ventilated metacrylate cylinder fitting the pot diameter and set up in a climate chamber in a complete randomized block design. Temperature and humidity inside the cylinders was recorded introducing USB dataloggers Log 32 (Dostmann electronic GmbH, Wertheim, Germany), on average the relative humidity was 76±2% inside the well-watered cylinders, and 73±2%, and 56±2% for mild and moderate drought conditions, respectively. The average temperature was 24±1°C in all cases.

The stress intensity of all plants at infestation and at each sampling time (4 or 10 dpi) was assessed by measuring: a) stomatal conductance (gs) and b) variations in maximum quantum yield of photosystem II photochemistry (Fv/Fm), as they are reported to be good drought indicators [2, 26]. Between sampling times, the stress status was not recorded to avoid mite disturbance. Stomatal conductance was measured using a leaf porometer (SC-1 Decagon-T, Pullman, USA) and the Fv/Fm was estimated using a FluorPen FP 100 (PSI, Drasov, Czech Republic). Both parameters were measured on the 4^th^ leaf, as preliminary experiments showed that it is representative of the plant status. Plant growth was estimated by measuring the stem length (distance between the soil and the terminal bud) and by weighting the aerial part of the plant (transformed to dry weight by using the water content data calculated as referred to below).

### Performance of *T*. *evansi* and leaf damage

The performance of *T*. *evansi* was determined by estimating mite population growth at 4 and 10 dpi. At each time, 15 infested plants per treatment were analyzed in the moderate stress experiment, and 9 infested plants per treatment in the mild stress one. All leaves were detached from the plant, and the number of eggs and mobile mite stages (larvae, nymphs and adults) were counted under a stereomicroscope M125 (Leica Mycrosystem, Wetzlar, Germany). To determine the leaf damage area (mm^2^ of chlorotic lesions), damaged leaflets were scanned using hp scanjet (HP Scanjet 5590 Digital Flatbed Scanner series, USA). The scanned leaflets were analyzed by the program GIMP 2.8 (www.gimp.org), for which we created a new layer, and the damaged area was painted in black color, then the number of pixels in black was counted using the histogram-tool. Finally, the number of pixels was transformed to mm^2^ using a scanned ruler as reference.

### Chemical and biochemical analysis of tomato plant material

Leaves from control and drought stress plants were collected and analyzed (6 plants/treatment). The collection was as follows: a) the left leaflets from leaves 3, 4 and 5 were collected together and frozen at -72°C; ground using a mortar and pestle in the presence of liquid nitrogen to a fine powder and stored for free amino acids, protein, protease inhibitors and oxidative enzymes analysis; and b) the right leaflets from the same leaves were collected together, dried in an oven at 70°C for 3 days, weighed before and after drying to assess the percentage of water and ground using a mortar and pestle to obtain a fine powder and stored for C, N and free sugar analysis. Unless otherwise specified, all chemical compounds used here and in the mite enzymatic activities assay were from Sigma-Aldricht (St Luis, USA).

#### Total C and N composition

Samples of 1 mg of dried leaf powder were analyzed to determine total nitrogen and carbon concentration at the Elemental Microelement Center of Complutense University (Madrid, Spain) by using a microelement analyzer LECO CHNS-932 (LECO, St Joseph, MI, USA).

#### Free sugars

Samples of 3 mg of dried leaf powder were homogenized in 650 μl of ethanol 95% (v/v), heated at 80°C for 20 min, centrifuged at 10000 rpm for 10 min, and the supernatant collected. The process was repeated two more times and the three supernatants were pooled. A volume of 750 μl of the mixture was dried on a SpeedVac Concentrator Savant SVC-100H (ThermoFisher scientific, Willmington, DE, USA) and redissolved in 500 μl of water. Soluble carbohydrate concentration was estimated by the anthrone method [27] using glucose as standard. In brief, 1 ml of anthrone reagent (0.2% v/v anthrone on 95% sulfuric acid) was added to the extract, heated at 90°C for 15 min, and the absorbance at 630 nm was measured using a VERSAmax microplate reader (Molecular Devices Corp., Sunnyvale, USA).

#### Free amino acids

The extraction of the free amino acids was done as described by [28]. Samples of 50 mg of leaf frozen powder were homogenized with 600 μl of water:chloroform:methanol (3:5:12 v/v/v). After centrifugation at 12000 rpm for 2 min, the supernatant was collected and the residue was re-extracted with 600 μl of the same mixture, pooling the two supernatants. A mixture of 300 μl of chloroform and 450 μl of water were added to the supernatants, and after centrifugation the upper water:methanol phase was collected and dried in a SpeedVac. The samples were dissolved on 100 μl of sodium citrate loading buffer pH 2.2 (Biochrom, USA) and 10 μl were injected on a Biochrom 30 Amino Acid Analyser (Biochrom, USA) at the Protein Chemistry Service at CIB (CSIC, Madrid, Spain).

#### Protein extracts

Samples of 100 mg of leaf frozen powder were homogenized in 500 μl of 0.15 M NaCl, ground with fine sand (Sigma, USA) in 1.5 ml tubes, the supernatant was frozen and stored at -20°C. The homogenate was centrifuged at 12000 rpm for 5 min at 4°C, and the total protein concentration was quantified using a Nanodrop 1000 spectophotometer (ThermoFisher scientific, Willmington, USA) with an absorbance ratio of A260/A280.

#### Protease inhibitors

The inhibitory activity of plant protein extracts was tested *in vitro* against commercial enzymes: papain (EC 3.4.22.2), cathepsin B from bovine spleen (EC 3.4.22.1), trypsin from bovine pancreas (EC 3.4.21.4) and α-chymotrypsin from bovine pancreas (EC 3.4.21.1). As substrates, Z-FR-AMC (N-carbobenzoxyloxy-Phe-Arg-7-amido-4-methylcoumarin) was used for papain, Z-RR-AMC (N-carbobenzoxyloxy-Arg-Arg-7-amido-4-methylcoumarin) for cathepsin B, Z-LA-AMC (Z-L-Arg-7-amido-4-methylcoumarin) for trypsin and SucAAPF-AMC (Suc-Ala-Ala-Pro-Phe-7-amido-4-methylcoumarin) for chymotrypsin, all of them purchased from Calbiochem (MerkMilipore, Billerica, USA). Samples of 20 μg of plant protein extracts were preincubated for 10 min with 100 ng of the commercial enzyme. The incubation was carried out at 28°C in 100 mM sodium phosphate buffer pH 6.0 (10 mM L-cysteine, 10 mM EDTA, 0.01% (v/v) Brij 35) for the papain and cathepsin B assays and at 35°C in 0.1 M Tris-HCl buffer, pH 7.5, for the trypsin and chymotrypsin assays. Subsequently, the substrates were added at a final concentration of 0.2 M and incubated for 1 h at 28°C or 35°C depending on the enzyme. Fluorescence was measured using an excitation filter of 350 nm and an emission filter of 465 nm (Tecan GeniusPro, Mannedorf, Switzerland). Double blanks were used to account for spontaneous breakdown of substrates and the plant protease activity, and all assays were done in duplicate. Results were expressed as a percentage of protease activity inhibited.

#### Oxidative enzymes

Samples of 100 mg of leaf frozen powder were ground in a 1.5 ml tube with sand and 500 μl of extraction buffer (0.1 M phosphate buffer, pH 7.0; 5% w:v polyvinylpolypyrrolidine). The homogenate was centrifuged at 12000 rpm for 10 min and the supernatant was collected and frozen until used. Polyphenol oxidase (PPO) activity was analyzed by incubating 20 μl of enzyme extract with cathecol (40 mM final concentration) in 160 μl of Tris-HCl pH 8.5 buffer at 30°C for 1 h. Absorbance was read at 420 nm. Peroxidase (POD) activity was determined incubating 20 μl of a 1:10 dilution of the enzyme extract with guaiacol (5 mM final concentration) and H_2_O_2_ (2.5 mM final concentration) in 150 μl of potassium phosphate pH 6 buffer at 30°C for 10 min. Absorbance was read at 470 nm. In both cases, a Varioskan Flash reader (ThermoFisher Scientific, Willmington, USA) was used.

#### Mite enzymatic activities

To analyze the effect of tomato drought-stressed plants on mite enzymatic activities an additional experiment was performed. Control and drought-stressed plants (6–7 leaves) were inoculated with 200 mites using the pump system previously described (6 replicates per treatment). Mites were collected at 10 dpi and frozen in liquid nitrogen. Mite protein extracts were obtained by homogenizing 1 mg of mites in 100 μl of 0.15M NaCl, centrifuged at 12000 rpm for 5 min and collecting the supernatant. Total protein content was determined according to the method of Bradford [29].

Cathepsin B-, cathepsin L- and legumain-like activities were assayed as described by [30], using Z-RR-AMC, Z-FR-AMC (Calbiochem, USA) and Z-VAN-AMC (N-carbobenzoxyloxy-Val-Ala-Asn-7-amido-4-methylcoumarin) (Bachem, Bubendorf, Switzerland) as substrates, respectively. The assays were performed by incubating 10 μl of mite protein extracts with 85 μl of 100 mM sodium citrate buffer (0.15 M NaCl, 1 mM DTT, pH 5.5 for cathepsin L- and B-like activities and pH 4.5 for legumain-like assay, containing 0.2 M of substrate) at 30°C for 15 min. Fluorescence was measured using an excitation filter of 350 nm and an emission filter of 465 nm on a Varioskan Flash reader (ThermoFisher Scientific, Willington, USA). A calibration curve was obtained with known amounts of AMC (7-amino-4-methylcoumarin) (Bachem, Swizerland).

Cathepsin D-like activity was determined using MocAc-GKPILFFRLK(Dnp)-D-R-NH2 (PeptaNova GmbH, Germany) as substrate The assays were performed incubating 5 μl of the mite protein extract with 85 μl of 100 mM sodium citrate buffer (0.15 M NaCl, 1 mM DTT, 10μM E-64, pH 3.5, containing 20 μM substrate) at 30°C for 15 min. Fluorescence was measured using an excitation filter of 328 nm and an emission filter of 393 nm on a Varioskan Flash reader (ThermoFisher Scientific, Willington, USA), and MCA (MoCAC-Pro-Leu-Gly) (Peptanova GmbH, Germany) was used as standard.

Leucine aminopeptidase-like activity was determined using LpNa (L-leucine p-nitroanilide) as substrate. Samples of 10 μl of mite extracts were incubated with 160 μl of 1mM LpNa in 0.1M Tris-HCl buffer (0.15M NaCl, 5mM MgCl2, pH 7.5) at 30°C for 4 h. The reaction was stopped with 100 μl of 30% acetic acid and the absorbance was measured at 410 nm, using a molar extinction coefficient of 8800 M-1 cm-1 for pNa.

α-Amylase activity was determined using starch as a substrate as described by [31]. Samples of 20 μl of mite extract were incubated with 80 μl of 0.1 M Tris-HCl buffer (40 mM CaCl_2_, 20 mM NaCl, pH 6.0) and 100 μl of 0.5% starch solution, at 30°C for 4 h. The reaction was stopped by adding 1 ml of lugol solution (0.02% I_2_; 0.2% KI). The mixture was centrifuged at 6000 rpm for 5 min and the supernatant’s absorbance was measured at 580 nm. A starch standard curve was used as a reference.

Esterase activity was determined using 1-naphthyl acetate (1-NA) as a substrate. Samples of 10 μl of a dilution 1/40 of the mite protein extract were incubated with 160 μl of 0.25 mM 1-NA in 0.1M Tris-HCl buffer (0.15M NaCl, 5mM MgCl2 pH 7.0) for 1 h at 30°C. The reaction was stopped by adding 80 μl of a solution (0.04% (w/v) of Fast blue salt BN (tetrazotized) and 1.5% (w/v) SDS) and incubating at room temperature for 1 h. A standard curve of 1-naphthol was used as a reference. Absorbance was measured at 600 nm.

Glutathione S-transferase (GST) activity was determined incubating 40 μl of mite protein extract with 220 μl of 0.1M Tris-HCl buffer (pH 8.0), containing 0.4 mM CDNB (1-chloro-2,4-dinitrobenzene) as substrate and 5 mM reduced glutathione as cofactor for 15 min at 30°C. The increment in absorbance at 340 nm was recorded every minute for 5 min, using a molar extinction coefficient of 9.6 mM–1 cm–1 for conjugated CDNB.

P450 activity was assayed by incubating 60 μl of mite protein extract with 120 μl of 100mM Tris-HCl buffer, (pH 7.0; containing the NADPH generating system [0.5 mM NADP, 2.5 mM glucose 6-phosphate and 0.3 units of glucose 6-phosphate dehydrogenase]) and 20 μl of a 50 μM cytochrome c solution at 30°C for 4 h. The reaction was stopped with 100 μl of methanol and the absorbance measured at 550 nm, using a molar extinction coefficient of 27.6 mM–1 cm–1 for cytochrome c reduced.

Hydrolytic activities were expressed as nmol of substrate hydrolyzed per min and mg of mite protein, GST activity as nmol of CDNB conjugated per min and mg of protein and P450 activity as nmol of cytochrome c reduced per min and mg of protein.

### Proline feeding stimulant test

Assays were performed in plastic Petri dishes (30x90 mm), coated in their bottom half with about 30 ml of a 0.625% agar solution to prevent leaf desiccation. The Petri dishes had a hole in the top, and were covered with filter paper to allow ventilation and preventing the escape of mites. Tomato leaf disks (3.14 cm^2^) were cut from well-watered tomato plants with a cork borer (Ø: 2 cm) and weighed. The abaxial and the adaxial surfaces of the leaf disks were dispensed with 25 μl of an ethanol solution of L-proline (Sigma, St Louis, USA) or ethanol alone for the control, and allowed to dry before mite inoculation. Concentrations were chosen to simulate the L-proline induced by both drought and *T evansi* infestation on tomato plants at 4 and 10 dpi (see Results), corresponding to 0.070 mg and 0.202 mg of L-proline/g of fresh leaf weight, respectively. Twenty *T*. *evansi* adult females from the colony, replicated ten times, were placed in each leaf disk (one per plate), being the number of eggs laid and the leaf damaged area recorded after 48 h. All Petri dishes were kept in a growth chamber (Sanyo MLR-350-H, Sanyo, Japan) at 25°C±1°C, 70±5% relative humidity and a 16 h light/8 h dark photoperiod.

### Statistical analysis

All plant and mite parameters analyzed were checked for the assumptions of normality and heteroscedasticity, and transformed if necessary. Stem length and stomatal conductance were log_10_(x) transformed, Fv/Fm was log_10_(x+1) transformed; and these two parameters and stem dry weight data were statistically analyzed using a three-way ANOVA (using as fixed factors drought treatment, *T*. *evansi* infestation and time). The number of *T*. *evansi* eggs and mobile forms and leaf damage area were log_10_(x) transformed and analyzed by a two way ANOVA (drought treatment, time). For stem length, plant aerial dry weight, stomatal conductance, Fv/Fm, mite population and leaf damage a Bonferroni *post hoc* test was performed to compare drought-stress treatments within each time. Percentage of nitrogen, protein, free amino acids and free sugars, as well as C:N ratio and protease inhibition were arcsen(squareroot(x)) transformed. These data and the oxidative enzyme activities were analyzed using a two-way ANOVA for each time separately using as fixed factors drought treatment and *T*. *evansi* infestation. A Newman-Keuls *post hoc* test was performed to see differences of mean between treatments. A Dunnet *post hoc* test was performed for every single amino acid data at 10 dpi, using as reference the control non-infested plant. Spider mite enzymatic activity data were analyzed by a t-student test. For the proline feeding assay, data were log_10_(x) transformed and analyzed by one-way ANOVA using proline concentration as a factor. A *Bonferroni posthoc* test was performed to determine the difference between treatments.

## Results

### Effects of drought on tomato plant growth, stomatal conductance and photosynthetic efficiency

**Fig 2:**
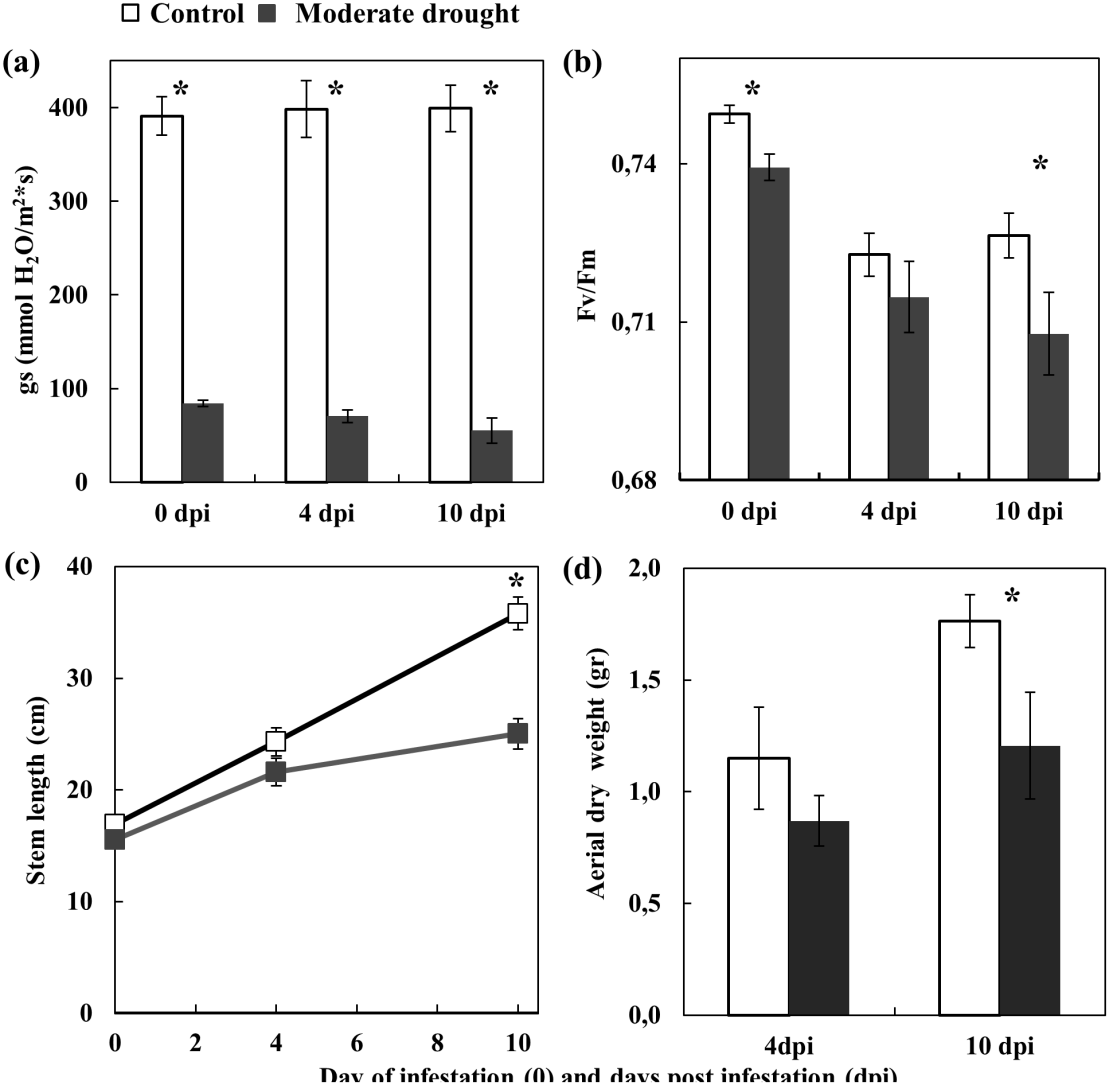
Effect of moderate drought at mite infestation and at 4 and 10 days post infestation (dpi) on: a) tomato stomatal conductance (gs); b) maximum quantum yield of PSII photochemistry (Fv/Fm); c) stem length; d) plant aerial dry weight. Data are mean ± SE. * indicates a statistically significant difference within each time (Three-way ANOVA, Bonferroni post hoc test, P<0.05).

Data from both infested and non-infested plants were pooled as no significant differences were found between them (data no shown). Moderate drought stress had a strong effect on stomatal conductance, which was significantly reduced between 4 and 5 times with respect to the control plants at 4 and 10 dpi (Fig 2a). The Fv/Fm of moderate drought stress plants was also significantly smaller than the control at infestation and at 10 dpi (Fig 2b). Similar effects, but less pronounced were obtained with mild stressed plants, with the stomatal conductance progressively reduced from about 20% less than in control plants at mite infestation to about half at 10 dpi, whereas Fv/Fm was only significantly lower at 10 dpi (S1 Fig). Significant differences in the growing pattern (aerial dry weight and/or stem length) were obtained at 10 dpi for both drought levels (Fig 2c, 2d & S1 Fig), with the reductions more acute for moderate stressed plants.

### Effects of drought on *T*. *evansi* population growth and leaf damage

**Fig 3:**
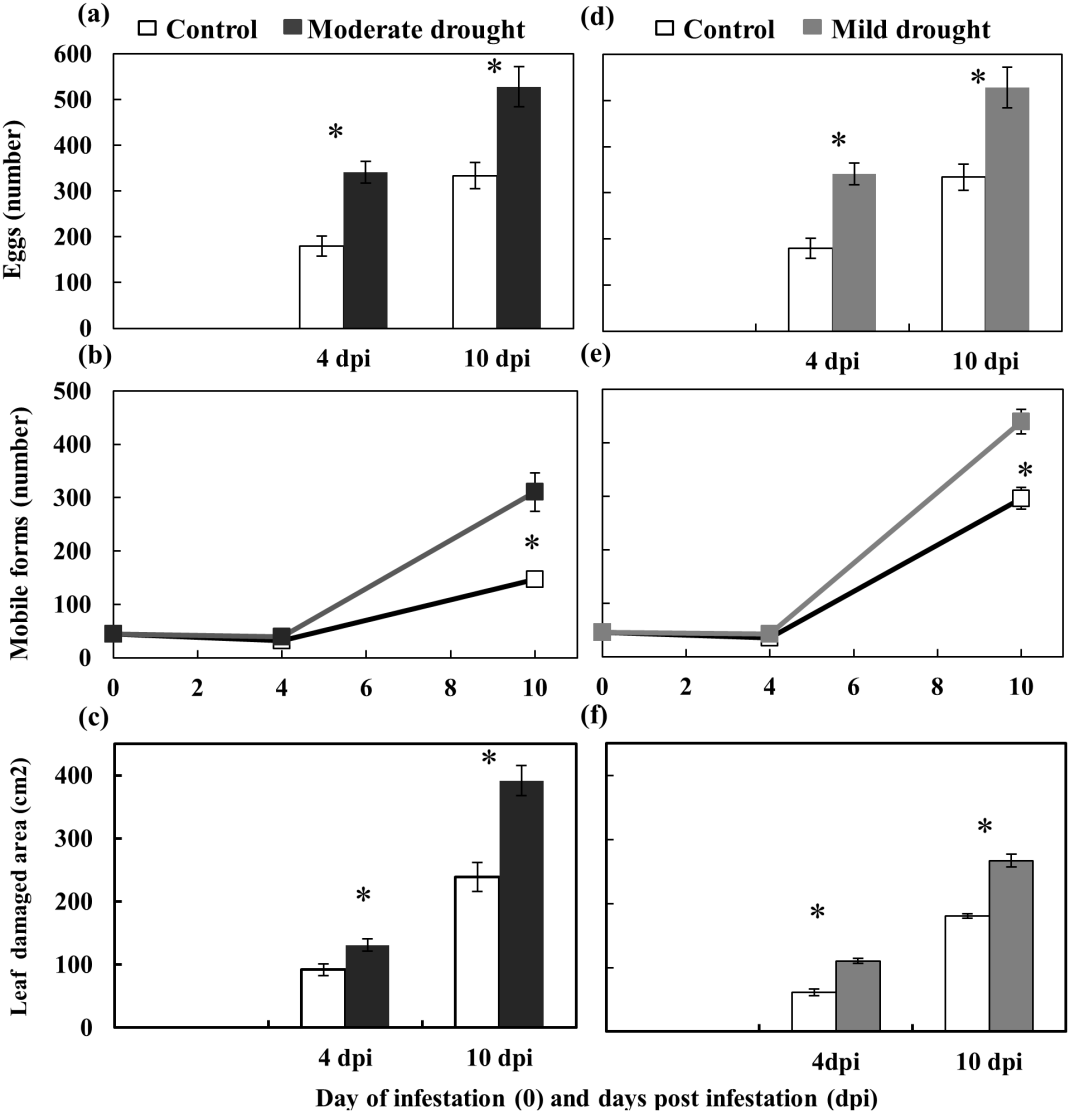
Performance of *T*. *evansi* in the moderate (a,b&c) and mild (d,e,f) drought stress experiments. The number of eggs (a&d), total mobile forms (b&e) and leaf damaged area (c&f) on control and drought stressed (mild or moderate) tomato plants at 4 and 10 days post infestation (dpi) were measured. Data are mean ±SE. * indicates a statistically significant difference within each time (Two-way ANOVA, Bonferroni *post hoc* test, *P*<0.05).

The population of *T*. *evansi* increased faster when feeding on both moderate and mild drought-stressed than on control tomato plants (Fig 3). The number of eggs laid under moderate stress was significantly higher at 4 (2 fold) and 10 dpi (1.6 fold) than on control plants (Fig 3a). No differences on mobile forms were observed at 4 dpi, as these individuals correspond to the surviving adult females (F0) that were used to infest the plant (Fig 3b). However, a significant increase (2.1 fold) was observed at 10 dpi, with most of these individuals from the resulting F1 progeny. Interestingly, the leaf damage area was 1.3 and 1.6 times significantly larger than on controls at both 4 dpi and 10 dpi, respectively (Fig 3c). When mites were reared on mild drought-stressed tomato plants, significant increases in eggs laid (Fig 3d), mobile forms (Fig 3e) and leaf damage area (Fig 3f) were also recorded.

### Effects of drought and *T*. *evansi* on plant nutritional composition and defense proteins

**Fig 4:**
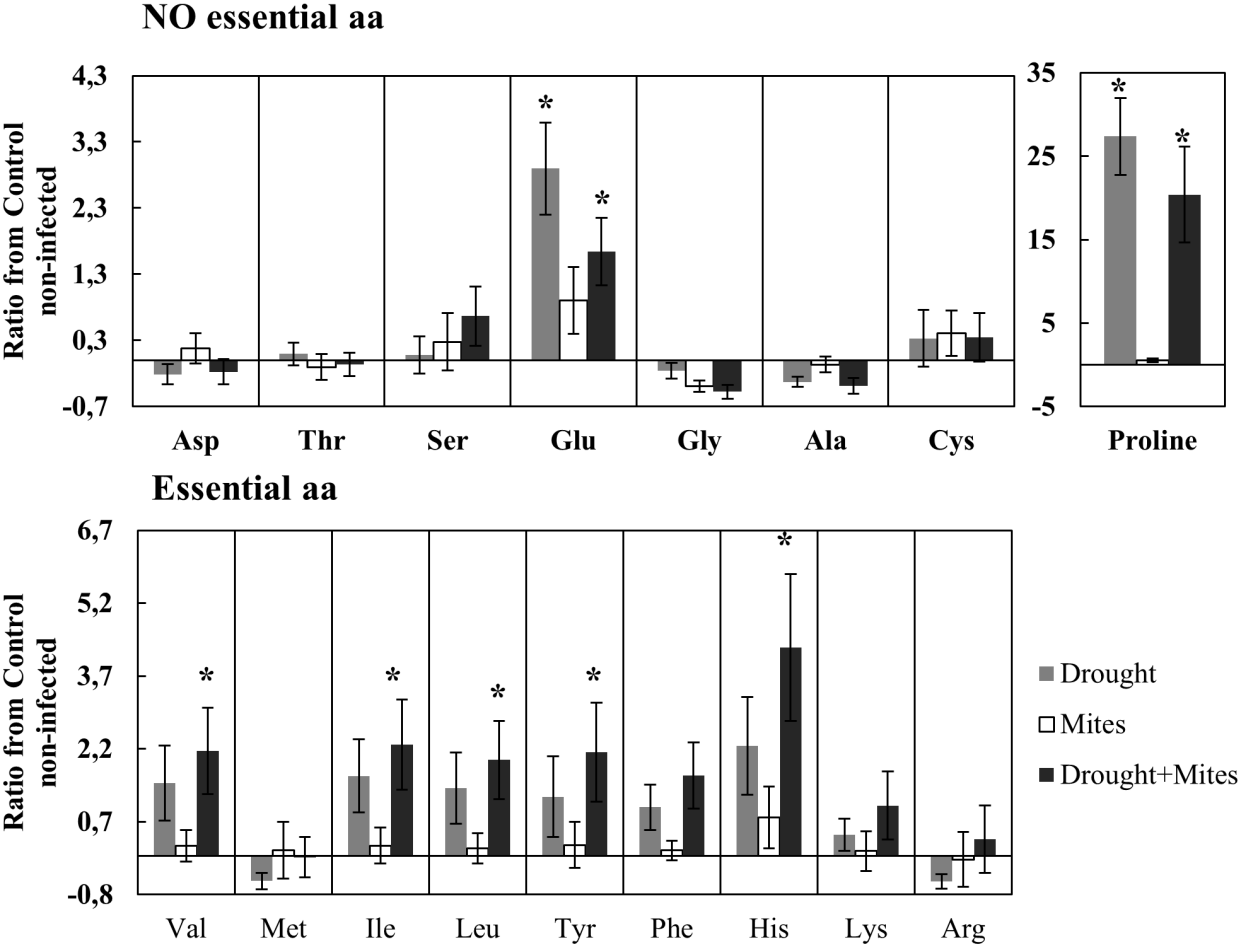
Levels of free amino acids in tomato leaves subjected to moderate drought stress and *T*. *evansi* infestation at 10 days post infestation (dpi). Data represent the ratio ± SE of the different treatments with respect to non-infested control plants at 10 dpi. = [(Treatment−Control 10dpi) / Control 10dpi]. The division between essential and nonessential amino acids is based on a study with the closely related species *Tetranychus urticae* [32]. * Indicates significant difference of the treatment with the control determined by Dunnet *post hoc* test p<0.05.

Differences in the levels of nutrients in the plants were found at 4 and 10 dpi, with drought stress the most significant factor (Table 1). At 4 dpi, drought, mite infestation and the combination of both factors produced a significant decrease of nitrogen and an increase of free sugars. Total protein was also reduced by drought at 4 dpi, but the difference was only significant in infested plants. At 10 dpi, a decrease in nitrogen and total protein, and an increase in free sugars were obtained with drought alone or in combination with mite infestation. Drought was also a significant factor for total free amino acids at 10 dpi, although no significant differences among treatments were found (Table 1). However, it could be demonstrated that drought drives the accumulation of specific amino acids (Fig 4). Proline was the amino acid more clearly induced by drought, increasing significantly by > 20 fold by drought alone or in combination with mite infestation (Fig 4). The levels of several essential (valine, isoleucine, leucine, tyrosine, histidine) and nonessential (glutamine) amino acids were also significantly induced by drought in combination with mites (Fig 4). The infestation by *T*. *evansi* did not have significant effects on any the amino acids analyzed at 10 dpi.

**Table 1:** Effect of moderate drought and *T*. *evansi* infestation on key nutrients in tomato leaves at 4 and 10 days post infestation.

	Non-infested		Infested		ANOVA (p<0.05)
	Control	Drought	Control	Drought	
**4 Days Post Infestation**					
**Nitrogen**^1^	6.7±0.1a	5.7±0.2b	5.8±0.2b	5.7±0.1b	D;I; D*I
**Protein**^1^	31±2ab	26±3b	35±2a	25±2b	D
**Free aa**^1^	1.0±0.2	1.2±0.2	1.0±0.1	1.1±0.2	-
**C:N**	5.9±0.1	6.4±0.2	6.5±0.3	6.3±0.2	-
**Free Sugars**^1^	3.3±0.1b	3.9±0.3a	4.2±0.1a	4.3±0.2a	I
**10 Days Post Infestation**					
**Nitrogen**^1^	6.3±0.2a	5.7±0.1b	5.9±0.2ab	5.3±0.1b	D
**Protein**^1^	40±5a	19±4b	27±6ab	15±2b	D
**Total free aa**^1^	0.9±0.2	1.7±0.3	1.2±0.3	1.8±0.4	D
**C:N**	6.3±0.1	6.4±0.1	6.1±0.2	6.6±0.2	-
**Free Sugars**^1^	3.2±0.2c	4.8±0.3a	3.7±0.1cb	4.3±0.3ab	D;D*I

^(1)^ Data, as % of dry weight, are mean ± SE. D (drought) and I (mites infestation) indicate significant factors in the Two-Way ANOVA. Different lower case letters within rows indicates significant differences (Newman-Keuls test at *p*<0.05).

Tomato plant defense proteins were also affected by drought stress and mite infestation, but different responses were obtained depending on the post-infestation time (Table 2). At 4 dpi, the inhibitory activity of tomato leaf extracts against papain was reduced by mite infestation under both drought and control conditions, whereas the inhibitory activity against chymotrypsin was reduced by drought conditions on non-infested plants. By contrast, the inhibitory activity of tomato leaf extracts at 10 dpi was enhanced against papain and cathepsin B as a response to both drought and mite infestation, whereas the inhibitory activity against trypsin increased in response to drought. The specific activity of the oxidative enzymes PPO and POD was significantly increased in response to mite infestation, drought stress and a combination of both at 4 dpi, but only in response to mite infestation at 10 dpi.

**Table 2:** Effect of moderate drought and *T*. *evansi* infestation on plant defense proteins in tomato leaves at 4 and 10 days post infestation.

	Non-infested		Infested		ANOVA (p<0.05)
	Control	Drought	Control	Drought	
**4 Days Post Infestation**					
**Protease inhibitors (% Inhibition)**					
Papain	48±2ab	64±2a	31±5c	45±5b	D;I
Cathepsin B	49±3	62±6	63±8	66±4	-
Trypsin	40±8	44±2	49±8	43±2	-
Chymotrypsin	61±7a	45±6b	64±2a	63±4a	I
**Oxidative enzymes (specific activity)**					
Polyphenol oxidases^1^	1.1±0.1b	2.1±0.1a	2.5±0.2a	2.6±0.3a	D;I;D*I
Peroxidases^2^	2.7±0.2b	5±0.5a	6.8±1.1a	5.1±0.2a	I; D*I
**10 Days Post Infestation**					
**Protease inhibitors (%Inhibition)**					
Papain	57±3c	81±3a	71±4b	86±3a	D;I
Cathepsin B	40±5c	60±7b	63±6b	83±5a	D;I
Trypsin	37±3ab	54±4a	31±8b	52±5a	D
Chymotrypsin	69±4	65±6	74±6	77±4	-
**Oxidative enzymes (specific activity)**					
Polyphenol oxidases^1^	2.2±0.1b	2.5±0.4b	3.7±0.5a	3±0.4ab	I
Peroxidases^2^	4.3±0.5b	4±0.5b	8.1±0.9a	5.8±0.6b	D; I

^(1)^ PPO: nmol hydrolyzed Cathecol/ mg Protein*min

^(2)^ POD: nmol hydrolyzed Guaiacol/ mg Protein*min.

Data are mean ± SE. D (drought) and I (mites infestation) indicate significant factors in the Two-Way ANOVA. Different lower case letters within rows indicates significant differences (Newman-Keuls test at *p*<0.05).

### Enzymatic activities of *T*. *evansi* fed on drought-stressed and well-watered tomato plants

Hydrolytic and detoxification enzyme activities in *T*. *evansi* were analyzed after feeding for 10 days on moderate drought-stressed or control tomato plants (Table 3). A significant increase in total mite protein and a reduction in the specific activity of cathepsin D- and legumain-like proteases and of α-amylase were observed on mites fed on tomato plants exposed to drought stress. The activity of the other digestive proteases (cathepsin B- and L-like and aminopeptidase) and detoxification enzymes (esterase, GST and P450) analyzed were not affected by the drought status of the plant in which they fed.

**Table 3:** Enzymatic activities in *T*. *evansi* feeding on moderate drought-stressed or control tomato plants for 10 days.

	Control	Drought-stressed
**Protein**^1^	55±2	69±5*
**Digestive enzymes (specific activity)**		
**Cathepsin B**^2^	7.8±1.0	8.7±1.1
**Cathepsin L**^2^	1.9±0.2	1.8±0.2
**Cathepsin D**^2^	13±2	5.5±0.4*
**Legumain** ^2^	0.29±0.03	0.20±0.02*
**Aminopeptidase**^2^	23±3	24±3
**α-Amylase**^2^	162±7	130±8*
**Detoxification enzymes**		
**Esterase**^2^	1774±146	1397±216
**GST**^3^	197±30	179±17
**P450**^4^	0.2±0.02	0.2±0.01

Data are mean ± SE.

* indicates statistically significant difference between treatments (t-student, p<0.05).

^(1)^ μg/mg fresh weight,

^(2)^ nmol substrate hydrolyzed per min and mg of protein,

^(3)^ nmol CDNB conjugated per min and mg of protein,

^(4)^ nmol cytochrome c reduced per min and mg of protein.

### Proline feeding stimulant assay

**Fig 5:**
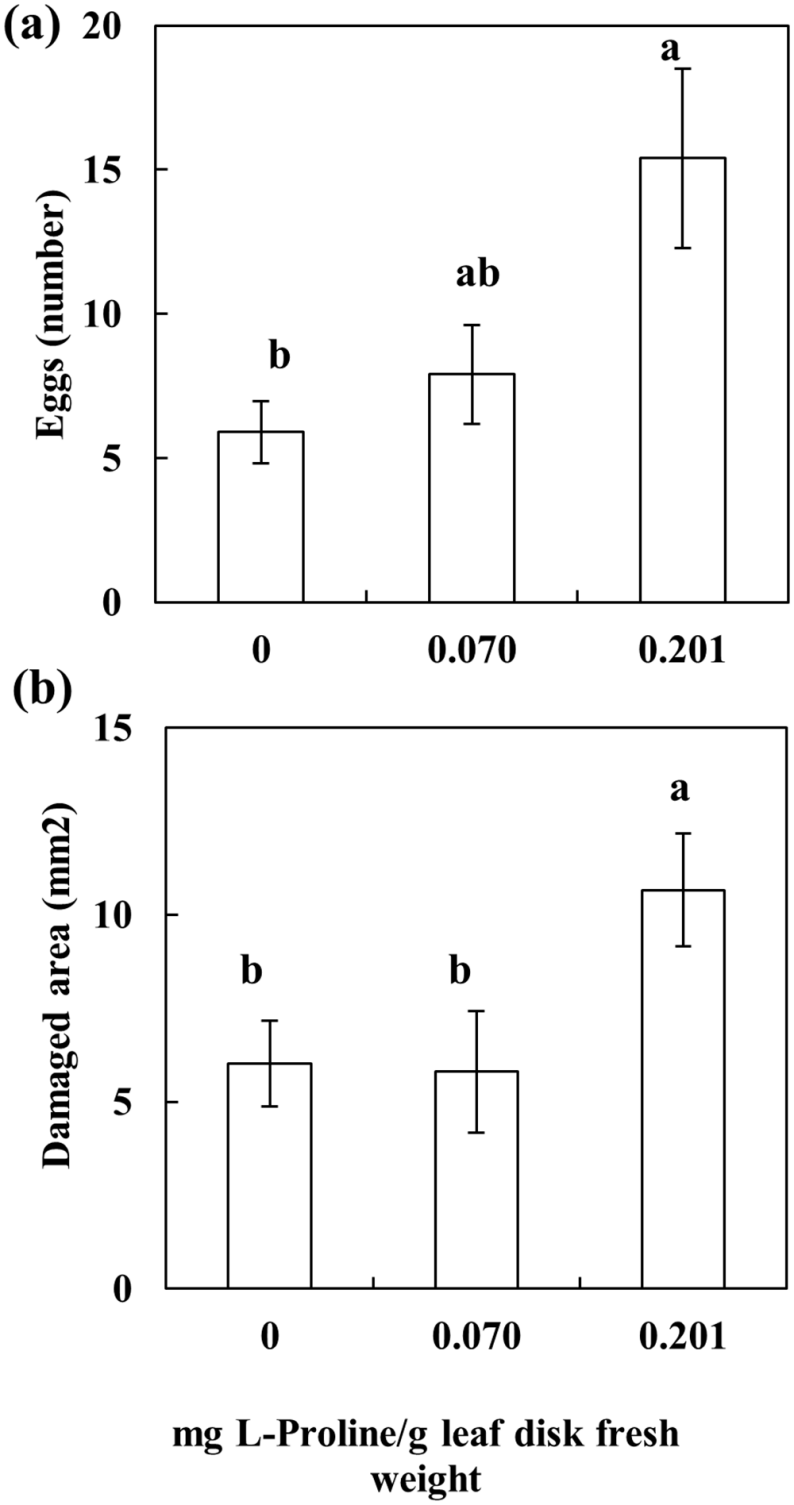
Performance of *T*. *evansi* with regard to the number of eggs laid (a) and leaf damaged area (b), on leaf disks treated with L-proline. Concentrations applied are equivalent to the one estimated on drought-stressed tomato plants at 4 and 10 days post infestation. Data are mean ±SE. Different letters indicate significant differences (One-way ANOVA, Bonferroni *post hoc* test, *P*<0.05).

The proline feeding stimulant assay showed a significant increase of eggs laid (2.5 fold) and leaf damage area (1.8 fold) on leaf disk treated with L-proline at the highest concentration tested (equivalent to that estimated for drought- and mite-stressed tomato plants at 10 dpi). In contrast, the effect was not significant when disks were treated with a concentration of proline equivalent to that estimated for drought- and mite-stressed tomato plants at 4 dpi (Fig 5). This result suggests a phagostimulant effect on *T*. *evansi* at the highest concentration of proline tested.

## Discussion

Under a climate change scenario, invasive mite species, such as *T*. *evansi*, raise further concerns because they might expand to new geographical areas [33] and have more generations per year, causing severe damage on tomato [34]. Our data revealed that mild and moderate drought-stressed tomato plants trigger bottom-up effects on *T*. *evansi*, with these plants physiologically more suitable for mite development. Mite performance was enhanced on both mild and moderate drought-stressed tomato plants, as revealed by a significant increase in the number of eggs laid and of mobile forms, resulting in more leaf damage. It has been reported that plants under drought stress mobilize proteins into amino acids and carbohydrates into free sugars for osmotic adjustments [7]. We have found that both drought and *T*. *evansi* damage triggered the decline of foliar soluble protein and total nitrogen content, suggesting that mites and drought are accelerating leaf senescence and likely inducing the transference of nutrients to younger leaves [35], as reported in cotton for the combined effects of water-stress and *T*. *urticae* infestation [36]. In contrast, the amount of several free amino acids and free sugars increased significantly in drought-stressed tomato leaves, as already reported for tomato [37], with proline the amino acid most clearly induced by drought. Interestingly, we have demonstrated that L-proline, a known phago-stimulant for insects [4, 24], also has a significant feeding stimulant effect on *T*. *evansi*, increasing both eggs laid and leaf damage area when added to tomato leaf discs. In addition, mite-infested, drought-stressed tomato plants showed significantly increased concentrations of five essential amino acids (valine, isoleucine, leucine, tyrosine and histidine), which have been reported to enhance the fecundity of the closely-related species *T*. *urticae* on strawberry, cucumber and chrysanthemum plants [38, 39]. Therefore, *T*. *evansi* is probably taking advantage of the increase in these plant-derived essential amino acids to synthesize structural proteins and enzymes, reducing the metabolic costs associated with digestion that can be reallocated for growth and development. A similar role can be proposed for the increase in free sugars, since it has been reported that fecundity of *T*. *urticae* correlated with sugar content in apple leaves [40], and free sugars can also act as phagostimulants for some herbivores [24]. Altogether, our data suggests that significant increases of available free sugars and essential amino acids, jointly with their phagostimulant effects, created a favorable environment for a better *T*. *evansi* performance on drought-stressed tomato leaves.

Another important aspect concerning the palatability of plants for mites is the level of anti-nutritional plant defense proteins that accumulate in response to abiotic and biotic stresses. As reported for other species [41], drought consistently induced the inhibitory activity of tomato-leaf extracts against cysteine (papain and cathepsin B) and serine (trypsin) proteases. This response was obtained for both *T*. *evansi* infested and non-infested plants, indicating that the response was driven by drought stress. By contrast, chymotrypsin inhibitory activity and the activity of PPO and POD varied depending on the time of plant exposure to drought stress, which may be a consequence of the complex regulatory pathways for some of these inducible defense genes in plants [42]. It has been reported that *T*. *evansi* suppresses plant defenses in tomato by down-regulating the induction of both the salicylic acid and jasmonic acid signaling pathways [43]. In particular, this was reflected in a reduction in the expression of serine protease inhibitors and PPO [20, 21], which are normally induced in tomato leaves in response to the attack by other spider mites [44]. Likewise, we have shown that the inhibitory activity against serine proteases (trypsin and chymotrypsin) was not significantly induced in leaves attacked by *T*. *evansi*, when compared to non-infested tomato plants. However, we found that mite infestation induced the inhibitory activity against cysteine proteases (cathepsin B and papain), which were not considered in previous studies. These results are especially relevant, since proteolytic digestion in spider mites relies on cysteine proteases (including *T*. *evansi*, see below) and cysteine protease inhibitors have been shown to be harmful against the spider mite *T*. *urticae*, in vivo [45]. We have also found that the specific activities of both PPO and POD were significantly increased in response to *T*. *evansi* infestation. These results may be considered somewhat contradictory with those obtained by Alba *et al*. [21], who reported that *T*. *evansi* suppressed the expression of two *PPO* genes (PPO-D and PPO-F) in tomato. However, this suppression only occurred when compared with tomato plants infested with mites from a non-suppressor *T*. *urticae* strain, but the expression of these genes in plants infested with *T*. *evansi* was slightly but significantly higher than on non-infested tomato plants [21] Thus, further studies to determine the *in vivo* effect of tomato cysteine protease inhibitors and PPOs on *T*. *evansi* are required.

Plant-feeding arthropods have developed a remarkable diversity of physiological adaptations to respond to changes in the nutritional composition of their host plants and to counteract the adverse effects of plant defenses including the regulation of digestive and detoxification enzymes [46, 47, 48]. The hydrolysis of dietary proteins in spider mites relies on cysteine (cathepsin B-, cathepsin L- and legumain-like) and aspartyl (cathepsin D-like) proteases and aminopeptidases [30, 49]. Our results indicate that *T*. *evansi* has a similar digestive proteolytic profile, hydrolyzing specific substrates for cathepsin D, cathepsin B, cathepsin L and legumain proteases and aminopeptidases. In addition, we determined the presence of α-amylase activity for the hydrolysis of carbohydrates in *T*. *evansi*, as already reported in other tetranychid mite species [50]. We found that cathepsin D- and legumain-like proteases and α-amylase activities were significantly reduced when *T*. *evansi* mites were fed on drought-stressed plants, but this reduction did not seem to affect their performance. The decrease of these mite hydrolytic activities could be explained by the ingestion of hydrolytic enzyme inhibitors from tomato plants. However, cathepsin B- and L-like activities in *T*. *evansi* were not affected, despite the induction of specific inhibitors for these two types of proteases in drought-stressed plants. These results suggest that *T*. *evansi* is capable of circumventing the adverse effects of at least some plant protease inhibitors. An alternative explanation for the reduction of some digestive protease and α-amylase activities in *T*. *evansi* could be related to the higher availability of nutrients in a readily assimilated form (essential amino acids and free sugars) in tomato plants subjected to drought conditions. Tomato plants are known to induce an array of secondary metabolites during periods of water deficit [23], thereby potentially increasing the concentration of compounds involved in plant defense. However, no significant changes in esterase, GST or P450 specific activities were observed when *T*. *evansi* was fed on drought-stressed plants. Secondary metabolites were not analyzed in this study, but it has been reported that *T*. *evansi* suppresses the expression of genes involved in regulation of secondary metabolites [21] and the release of inducible volatiles [20]. This down-regulation of plant defenses may render unnecessary the induction of the mite detoxification enzymes, and the metabolic resources saved diverted toward growth and reproduction. Nevertheless, further studies will be needed to determine the combined effects of drought and *T*. *evansi* infestation in the regulation of secondary metabolites in tomato.

## Conclusions

Our data reveal that both drought and *T*. *evansi* infestation induced significant changes in the nutritional quality of tomato plants, as more essential amino acids and free sugars are available. These changes trigger a bottom-up effect on key biological traits of *T evansi* causing a highly significant increase of leaf damage and mite performance. Plant defense proteins were also induced by drought and/or mite infestation, but the mite physiological responses suggest that *T*. *evansi* is capable of circumventing their potential adverse effects. These findings support the “Plant stress hypothesis” and suggest that drought-stressed tomato plants even at a mild level may be more prone to *T evansi* outbreaks in a climate change scenario, which might strongly affect tomato production on area-wide scales. Moreover, abiotic stresses can also exert a strong impact at higher tropic levels. Thus, it has been demonstrated recently that plant water status may also trigger bottom-up effects on the reproductive behavior and fitness of some natural enemies, especially the omnivorous predators [51, 52], which might shape the arthropod structure. Therefore, this complexity of food-web structures should be considered when using species distribution models to infer climate change effects on crop pests.

## Supporting Information

S1 Fig Effect of mild drought at mite infestation and at 4 and 10 days post infestation (dpi) on: a) tomato stomatal conductance (gs); b) maximum quantum yield of PSII photochemistry (Fv/Fm); c) stem length; d) plant aerial dry weight. Data are mean ± SE. * Indicates significant difference within each time (Three-way ANOVA, Bonferroni *post hoc* test, *P*<0.05).
(TIF)
