# Supplementary figures and images for: Drought-Stressed Tomato Plants Trigger Bottom–Up Effects on the Invasive Tetranychus evansi
Source: PLoS One. 2016 Jan 6;11(1):e0145275. doi: 10.1371/journal.pone.0145275 (PMC4703393; doi:10.1371/journal.pone.0145275)

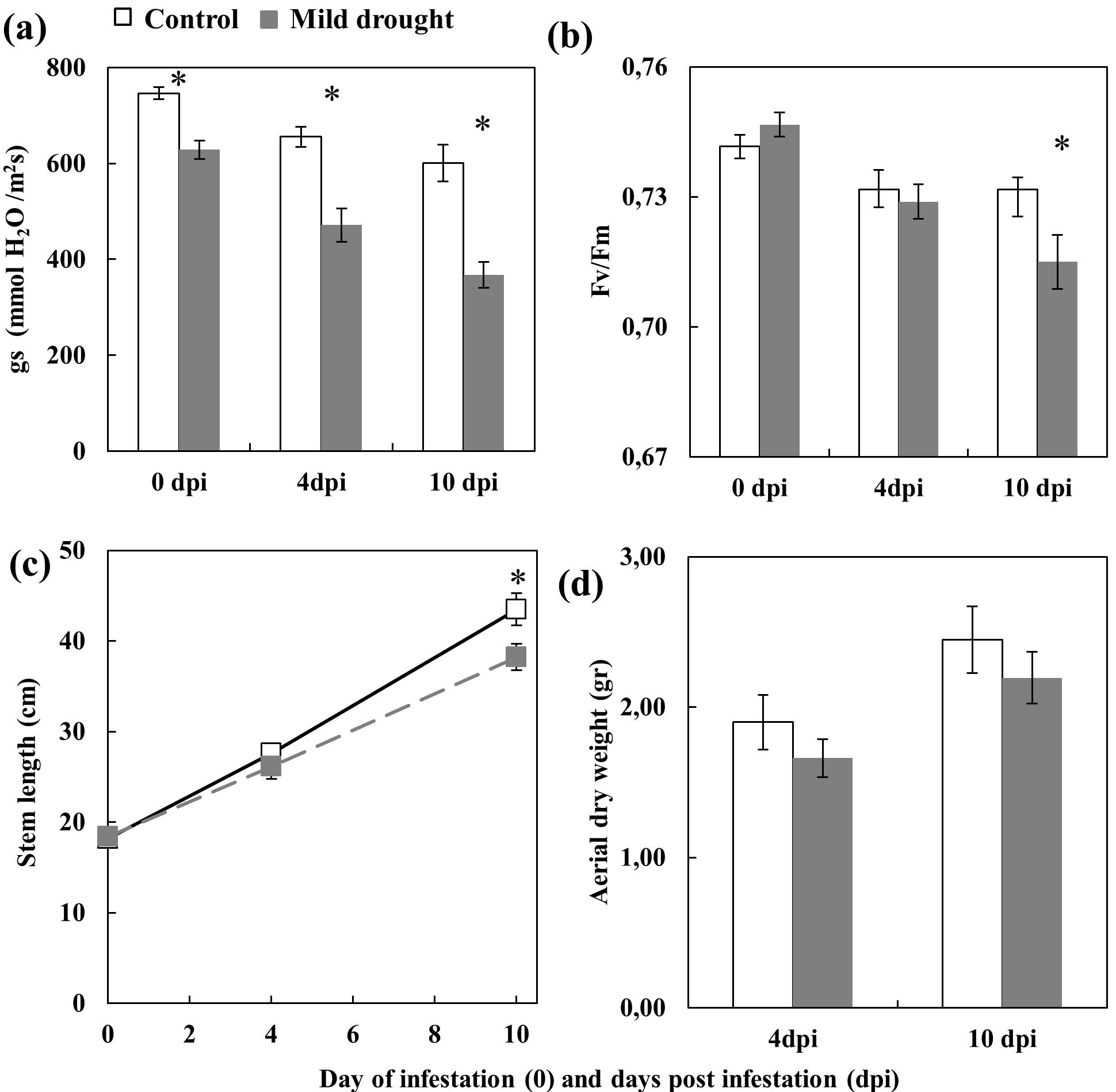

Supplement: S1 Fig — Data are mean ± SE. * Indicates significant difference within each time (Three-way ANOVA, Bonferroni post hoc test, P<0.05). (TIF) [file pone.0145275.s001.TIF]
